# From labs to field realities: a paradigm shift in rice salinity screening

**DOI:** 10.3389/fpls.2026.1812738

**Published:** 2026-04-22

**Authors:** Joie Ramos, Mahender Anumalla, Margaret Catolos, Ma Teressa Sta. Cruz, Sankalp Bhosale, Waseem Hussain

**Affiliations:** 1 Rice Breeding Innovation Platform, International Rice Research Institute (IRRI), Los Baños, Laguna, Philippines; 2 International Rice Research Institute (IRRI)‐South‐Asia Hub, International Crops Research Institute for the Semi-Arid Tropics (ICRISAT), Hyderabad, Telangana, India

**Keywords:** rice, salinity, phenotyping, controlled conditions, fields, seawater

## Introduction

Rice (*Oryza sativa* L.) is a cornerstone of global food security, providing a primary dietary energy source for over 3.5 billion people and accounting for approximately 20% of global caloric intake (Wing et al., 2018). As the global population is projected to reach 10 billion by 2050, the demand for rice is expected to intensify, particularly in Asia and Africa, which are already facing significant yield constraints due to salinity stress (Zheng et al., 2023; Clay et al., 2024). Coastal and deltaic rice-growing areas of Asia, which collectively account for 65% of global rice production, are increasingly vulnerable to saltwater intrusion driven by sea-level rise, tidal surges, and climate-induced hydrological changes (Wassmann et al., 2009; Al-Tamimi et al., 2016). Salinity levels exceeding 4 dS/m (decisiemens per meter) can reduce rice yields by more than 50%, posing a severe threat to food production in these ecologically fragile areas (Mohanavelu et al., 2021; Melino and Tester, 2023).

Developing salt-tolerant rice varieties is widely recognized as the most sustainable and cost-effective strategy to mitigate salinity-induced yield losses (Khanna et al., 2024). National and international breeding programs have made notable progress in this direction (Ismail and Horie, 2017). In parallel, advances in molecular genetics and genomics have led to the identification of numerous salinity-responsive genes and quantitative trait loci (QTLs) (Ismail and Horie, 2017). However, translating these discoveries into elite, farmer-preferred cultivars has been largely unsuccessful (Hu and Schmidhalter, 2023). Furthermore, despite the availability of promising genetic resources, their deployment in breeding pipelines has not led to widespread adoption or impact at the farm level (Melino and Tester, 2023). A significant constraint underlying this translational gap is the continued reliance on phenotypic screening under highly controlled, idealized conditions-typically in hydroponic systems, greenhouses, or growth chambers (Mittler, 2006; Munns et al., 2006; Araus and Cairns, 2014; Gilliham et al., 2017; Negrão et al., 2017; Liu et al., 2022; Rawat et al., 2022).

To accelerate the development of salinity-tolerant rice varieties and improve the translational efficiency of genomic discoveries, a fundamental shift in phenotyping strategy is required (Tracy et al., 2020; Moshelion et al., 2024). In this viewpoint, we introduce a novel field-based phenotyping platform specifically designed to overcome the limitations of conventional, controlled-environment screening. This platform enables the evaluation of rice genotypes under ecologically realistic salinity stress conditions that closely mimic those encountered in farmers’ fields. Crucially, it allows the identification of genotypes with stable performance under complex field conditions, traits that are often overlooked in reductionist settings. This approach represents a critical advancement in bridging the gap between laboratory discovery and field application, offering a scalable and scientifically rigorous pathway to deliver resilient, high-performing rice cultivars to salt-affected regions.

## Ideal isn’t enough: real-world salinity screening matters

The continued reliance on idealized screening environments has significantly constrained the development of salt-tolerant rice varieties. Traditionally, salinity stress evaluation in controlled conditions has primarily been used to facilitate gene discovery, QTL mapping, and mechanistic studies (Mittler, 2006; Hu and Schmidhalter, 2023). The rationale behind controlled screening lies in its ability to minimize environmental noise, such as soil heterogeneity and fluctuating stress levels, thereby enhancing the reproducibility and precision in identifying genetic loci (Tracy et al., 2020; Moshelion et al., 2024).

The concept of environmental reductionism—the simplification of growing conditions to isolate genetic effects—has dominated abiotic stress research, including salinity screening in rice (Plessis, 2023). While these controlled systems have been instrumental in elucidating the physiological and molecular mechanisms of salinity tolerance (Mittler, 2006; Heil and Schmidhalter, 2017), they fail to capture the dynamic, multifactorial variable conditions that define real-world agroecosystems (Cominelli et al., 2013; Deinlein et al., 2014).

Salinity in farmers’ fields is neither uniform nor static; it varies spatially and temporally due to factors such as rainfall, irrigation, evapotranspiration, and soil composition (Valenzuela et al., 2022). Consequently, genotype performance under controlled conditions often does not correlate with field performance, which limits the predictive power of selection and the translational value, thereby hindering the development of resilient cultivars (Plessis, 2023). This disconnect raises critical concerns about the utility of such approaches for breeding complex traits, such as salinity tolerance.

To address this bottleneck, a significant change is necessary —one that facilitates field-based phenotyping under realistic conditions. This transition is crucial for enhancing the application of genetic discoveries to develop resilient, high-yielding rice varieties that can thrive in saline-prone agroecosystems.

## A paradigm shift: embracing realistic field-based screening

Rice salinity research stands at a critical crossroads. Success now depends on abandoning idealized lab and greenhouse screenings in favor of field-based evaluations that reflect the true complexity of farmers’ environments. This shift is not optional; it is essential for identifying genotypes that can withstand the unpredictable, multifactorial nature of salinity stress in real-world conditions.

Field-based screening offers several key advantages: A) It reflects the dynamic nature of saline agroecosystems, enabling the identification of genotypes with stable and reliable performance; B) It allows for a more accurate assessment of genotype adaptability and resilience across spatial and temporal gradients; and C) It provides deeper insights into trait expression and the genetic architecture underlying salinity tolerance and yield stability.

Despite these advantages, field-based screening presents significant challenges. Soil salinity heterogeneity, spatial variability in the field, and temporal fluctuations resulting from rainfall or irrigation introduce environmental noise, which complicates genotype evaluation and gene identification (Valenzuela et al., 2022). These complexities have historically discouraged researchers from adopting field-based salinity screening approaches. To address these challenges, it is essential to develop robust field protocols that manage salinity variability and maintain consistent stress levels across key growth stages.

At the IRRI (International Rice Research Institute), 1996, we have developed and optimized a field-based salinity screening platform that simulates real-world conditions. This also includes the world’s first seawater-based salinity screening facility, specifically designed to replicate the challenges of coastal rice-growing ecologies (https://bit.ly/3SQiJiP).

## Features of the new salinity screening setup

**Figure 1:**
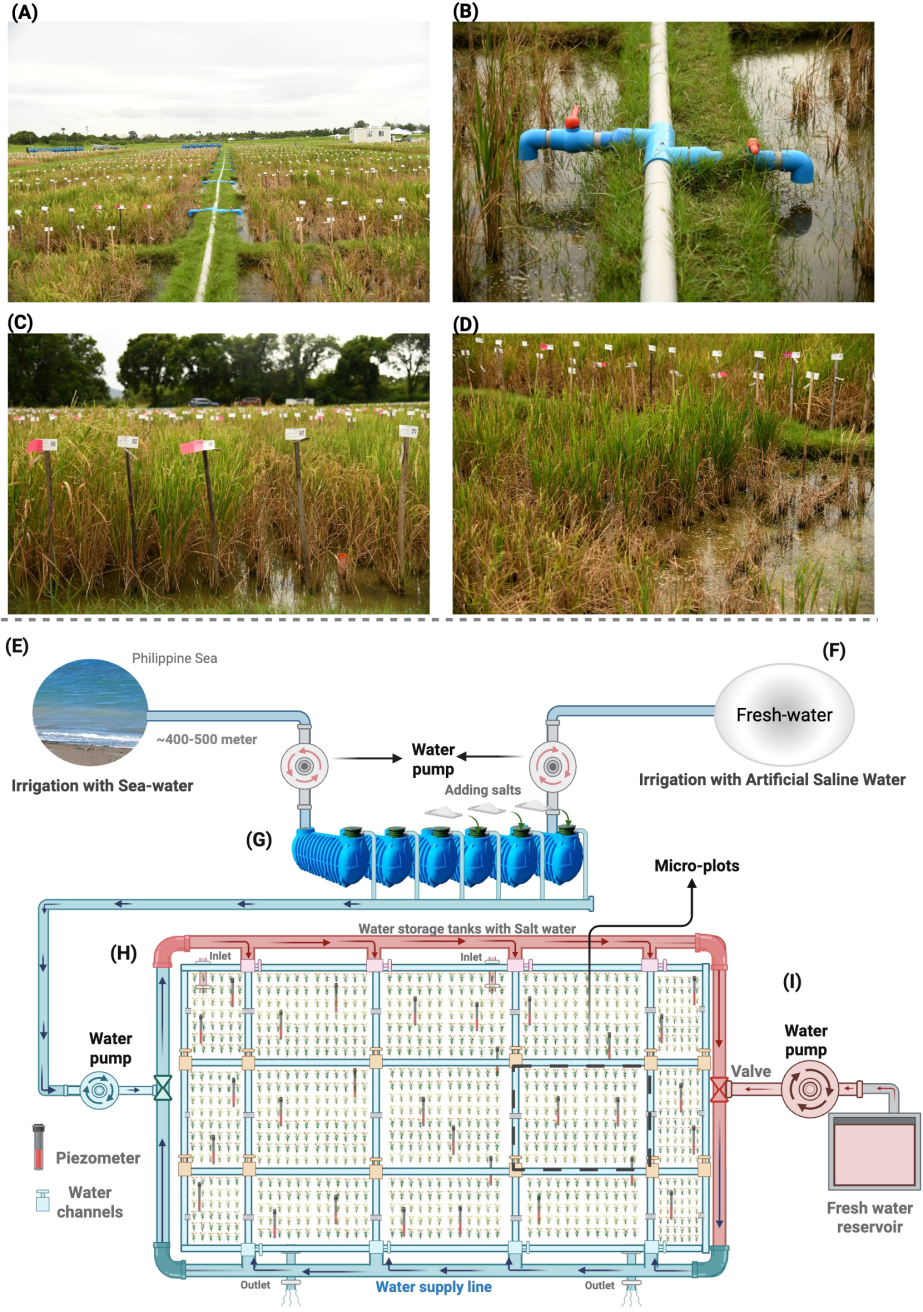
Illustration of the new field-based phenotypic salinity screening setup at IRRI-HQ. **(A)** It depicts the layout, showing how the field is divided into small microplots with water-houses featuring inlets that connect to each microplot for saline-water irrigation; **(B)** provides a close-up image of the inlets, which control irrigation for each microplot; and **(C, D)** display the visualization of the genotypes in the field, demonstrating the effects of salinity and how we can easily differentiate among the genotypes in the field with the new setup. In **(E)**, seawater is drawn from approximately 300 meters off the coast of the Philippine Sea and stored in water tanks in new salinity field screening setup; In **(F)**, freshwater is pumped from a reservoir and stored in tanks, where salts are added in the correct concentrations to achieve the desired electrical conductivity (EC); **(G)** The salinized water is then distributed through a network of hoses across the field. Each hose has controlled inlets and outlets, with each microplot containing 1–2 inlets for irrigation; **(H)** The hoses connected to each microplot have water flows controlled by gated water channels. Piezometers are installed in every microplot to monitor EC levels; **(I)** If salt concentration is too high, fresh water is used to dilute the salinized water, particularly in naturally hot conditions, to reach the desired EC. The field layout ensures that drainage canals surround each microplot, allowing for controlled water drainage through outlets into the main canals.

The newly developed field-based salinity screening setup addresses long-standing challenges in realistic phenotyping—specifically, soil salinity heterogeneity, spatial variability, and the need for precise stress control across growth stages (**Figure 1**). This setup represents a significant intervention in that direction. A detailed protocol is provided in **Supplementary File S1**. Below are the key features that make this system innovative, scalable, and impactful.

### Minimizing soil salinity heterogeneity

The setup incorporates three core design elements to manage soil salinity and reduce spatial variability: a) microplot layout, b) saline-water irrigation using pumps, and c) row and column coordinates for spatial correction. In the micro-plot concept, the setup divides the field into small, uniformly sized microplots (typically 6 × 6 m or 36 m²), arranged systematically to allow high-resolution control of salinity stress (**Figure 1**). This design enables precise irrigation and stress management at the plot level, which is far more effective than managing large field blocks. Each microplot is carefully levelled and pre-conditioned with fresh water to establish a uniform baseline electrical conductivity (EC) before transplanting. Depending on the experimental design, 5–6 microplots can be accommodated per block.

Instead of manually applying salts to the soil—a labor-intensive and inconsistent method—salts are dissolved in tanks and applied via water pumps for precision irrigation with saline water (**Figure 1**). Each microplate is individually irrigated, ensuring rapid and uniform distribution of saline water. For example, an entire field of microplates can be irrigated in just 20–30 seconds, minimizing variability in salt exposure and improving reproducibility (**Figure 1**). The concentration and amount of salt added to the tanks have been optimized. For example, the salt concentration added to a 20-litre tank is 25 kg to achieve the EC of 30 dS/m. We recommend piloting this approach because it will largely depend on the type and concentration of salt being used.

**Figure 2:**
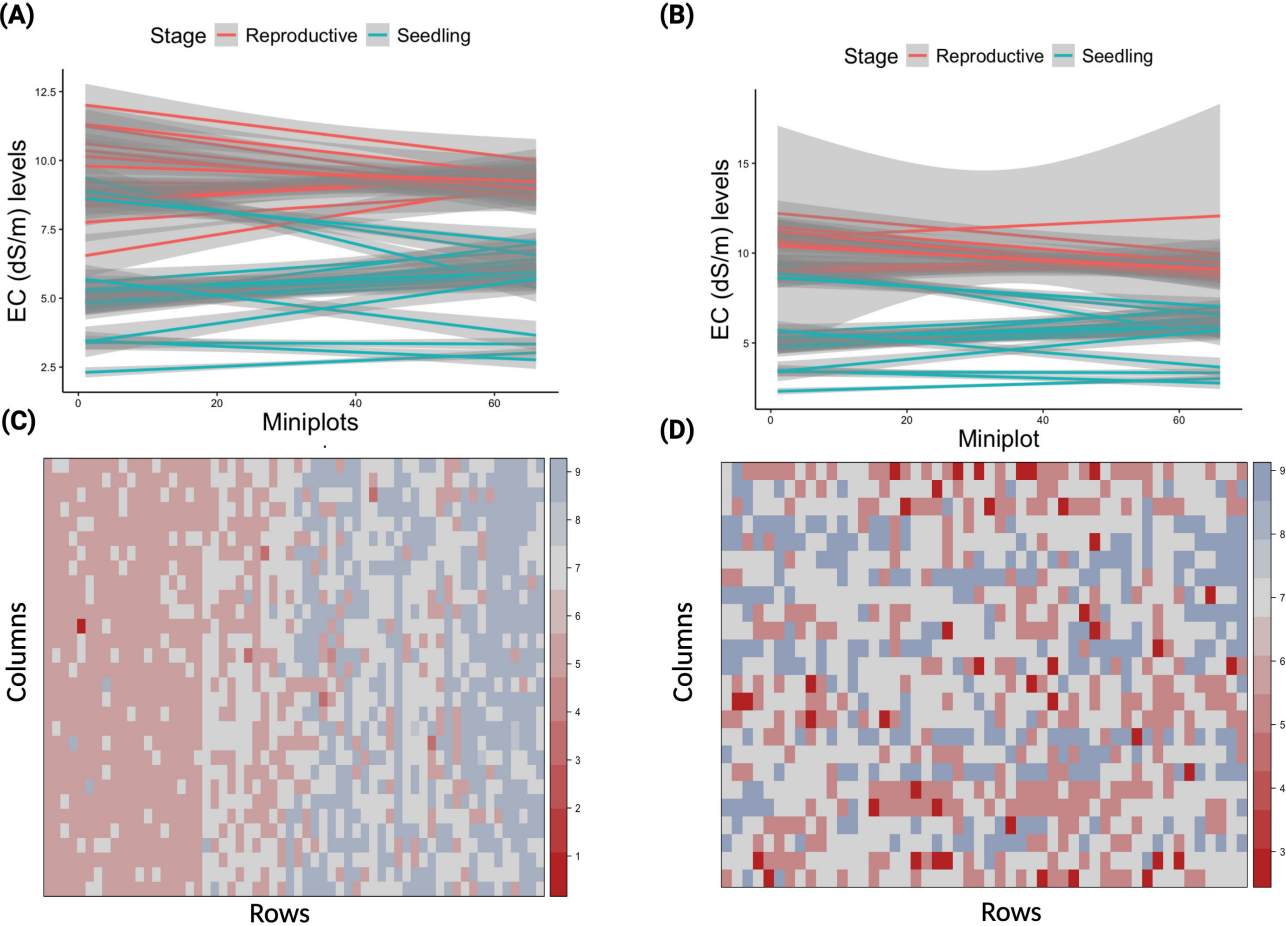
Illustrates the maintenance of EC thresholds and spatial variability in the salinity field at IRRI-HQ. **(A)** shows a line plot of EC levels across microplots during seedling/vegetative and reproductive stages in the original setup, where the non-parallel lines indicate inconsistent EC distribution across microplots; **(B)** presents the same field under the new setup, where parallel lines reflect consistent EC levels across microplots. The gray bars represent the standard error in EC levels across the growth stages; **(C)** displays a heatmap of Standard Evaluation Scores (SES) in the original field, with scores ranging from 1–9: dark red (1–3) indicates high tolerance, light red (4–5) intermediate, and light blue (6–9) low tolerance. The clustering of tolerant genotypes on one side and sensitive ones on the other highlights significant spatial heterogeneity and variability in the field, and **(D)** shows the SES score heatmap under the new setup, where the randomized distribution of scores and colors suggests minimal spatial variability, demonstrating the effectiveness of the new salinity screening setup in reducing field heterogeneity.

In addition to microplot design and precision saline water irrigation, each microplate and block uses row and column coordinates (**Figure 2**). This spatial mapping enables the use of mixed-model frameworks to correct for soil variability using EC measurements collected throughout the experiment. These models significantly enhance the accuracy of genotype performance estimates (Bazihizina et al., 2012; Longo et al., 2020; Tano et al., 2020; Adhikari et al., 2022; Bahjaouy et al., 2025). More details on mixed models accounting for spatial trends are available in our analytical pipeline (Hussain et al., 2022).

To substantiate the claim that this platform represents a significant advancement over existing field-based salinity screening approaches, we present comparative evidence from our trials at IRRI. The heatmaps of Standard Evaluation Scores (SES) in **Figure 2C** (original setup) and **Figure 2D** (new setup) provide direct visual evidence of the improvement (International Rice Research Institute, 1996). In the original setup (**Figure 2C**), tolerant genotypes (dark red, SES 1-3) cluster on one side of the field while sensitive genotypes (light blue, SES 6-9) cluster on the other, indicating that spatial heterogeneity in the field—not true genetic differences—was driving phenotypic scores. In contrast, the new setup (**Figure 2D**) shows a randomized distribution of SES scores across the field, confirming that the microplot-based design, precision saline-water irrigation, and spatial correction models effectively minimize spatial variability and enable unbiased genotype evaluation.

#### Evidence of improved phenotyping precision and genotype discrimination

To provide rigorous quantitative evidence of the platform’s enhanced phenotyping performance, we compared variance components and broad-sense heritability estimates derived from mixed-model analyses (Hussain et al., 2022) of SES scores under the original and redesigned field-based salinity screening setups. In the original configuration, the residual variance (σ^2^_e_) and the variance associated with replicates nested within microplots (σ²_{Rep: microplot}) were disproportionately large relative to the genotype variance component (σ²_G). This imbalance suppressed the proportion of phenotypic variance attributable to genetic effects, resulting in a low broad-sense heritability (*H²* = 14.50%). Such a variance structure is indicative of substantial uncontrolled spatial heterogeneity and microenvironmental noise, which compromise genotype discriminability.

In contrast, the new screening platform substantially altered the partitioning of phenotypic variance. The residual variance decreased by 25.21%, reflecting improved control of fine-scale environmental variability and more consistent stress imposition across microplots. Simultaneously, the genotype variance increased by 72.55%, suggesting that the redesigned layout more effectively exposes and captures genetic differentiation in salinity response. Although the nested block variance increased (+111.7%), the relative increase in σ²_G was greater, leading to an overall improvement in the signal-to-noise ratio. Consequently, the heritability increased to H² = 23.89%, representing a 64.8% gain over the original setup (**Table 1**). This increase in *H²* demonstrates that the new platform reduces experimental noise and enhances the precision with which genotypic differences in stress response are estimated—an essential attribute for reliable genetic selection. These quantitative improvements align with the observed reduction in spatial variability in the geostatistical heatmaps (**Figures 2C** vs **2D**), confirming that the redesigned field setup systematically enhances the stability, reproducibility, and discriminatory power of phenotyping under salinity stress.

**Table 1:** Comparison of variance components and broad-sense heritability (*H²*) for Standard Evaluation Scores (SES) between the original and new field-based salinity screening setups at IRRI.

Variance component	Original setup	New setup	Change (%)
Genotype variance (σ²_G)	0.290	0.500	+72.55%
Rep: Block variance (σ²_R:B)	0.231	0.489	+111.7%
Error variance (σ²_E)	1.477	1.104	−25.21%
Broad-sense heritability (H²)	14.50%	23.89%	+64.8%

### Precise control over salinity thresholds

Maintaining consistent salinity stress levels—spatially and temporally—is essential for reliable phenotyping. The seedling and reproductive stages are susceptible to salinity, and precise control during these phases is critical for identifying robust genotypes (Kong et al., 2017; Sahab et al., 2021; Atta et al., 2023; Mheni et al., 2024; Oelviani et al., 2024). We utilize piezometers and a flushing-out strategy, employing fresh water, to control and precisely measure salinity thresholds in the setup (**Figures 1E–I**).

Furthermore, the uniformity of salinity stress across growth stages is a key advantage of this platform. Unlike the original approach of manually spreading salt granules in the field—which is labor-intensive, time-consuming, and inherently inconsistent—the new setup uses pre-dissolved saline water delivered through a network of PVC pipes and water pumps. As illustrated in **Figure 2A** (original setup), the non-parallel EC lines across microplots indicate inconsistent EC distribution, reflecting variable stress levels across the field. In contrast, **Figure 2B** (new setup) demonstrates highly parallel EC lines, indicating uniform salinity stress across all microplots at both the seedling/vegetative and reproductive stages. The standard error bars in **Figure 2B** are notably smaller compared to **Figure 2A**, confirming reduced within-stage variability. This uniformity is critical because it ensures that all genotypes are evaluated under comparable stress conditions, thereby enabling reliable genotype ranking and selection.

Piezometers (small plastic tubes measuring 30 cm in length and 2 inches in diameter PVC pipe) are installed in each microplot (typically 4–6 per 36 m² plot) to monitor EC levels (Portable High Range EC/TDS Meter - HI99301P) throughout the crop cycle (**Figures 1E–I**). The piezometers are critical as they measure the EC of the root zone, which is more crucial for evaluating the stress faced by the plants. EC is calculated daily using a Fisher EC meter, with target thresholds maintained below 10 dS/m during the seedling stage and between 12 and 15 dS/m during the reproductive stage (**Figure 1**).

A flushing strategy is employed to manage excessive salinity, especially when using seawater (50–60 dS/m). Fresh water is introduced through dedicated canals and inlets to reduce EC to the desired range (15–20 dS/m) (**Figure 1**). This approach ensures that salinity levels remain within optimal thresholds, allowing for the accurate evaluation of the genotype (**Figure 2**).

### Scalable, reliable, and field-relevant phenotyping

Traditional salinity screening methods have long been constrained by low throughput, high labor demands, and limited scalability—factors that have hindered the consistent evaluation of large plant populations. Large saline field trials have been rare due to challenges in managing soil heterogeneity and environmental variability. Moreover, the lack of reliable phenotyping under realistic field conditions has been a significant barrier to breeding for salinity stress (Hu and Schmidhalter, 2023).

The newly developed screening setup addresses these limitations by providing a high-throughput, cost-effective, and field-relevant platform. For example, at IRRI-HQ, up to 3,500 genotypes (2–3 m² plot size) can be screened per season, while at the Infanta site in the farmer’s field using seawater irrigation, approximately 3,000 entries can be evaluated. Its modular design allows for easy replication across diverse agroecological zones, making it adaptable to real-world salinity conditions.

Beyond scalability, the setup enables robust and reproducible phenotyping by integrating precise salinity control, spatial correction models, and microplot-based design (Heil and Schmidhalter, 2017). This allows the accurate quantification of soil heterogeneity and salinity dynamics—factors crucial for identifying genotypes with genuine agronomic value (**Figure 2**). The platform is particularly well-suited for early-generation selection, trait dissection, and genetic studies, providing a powerful tool for breeding programs seeking to develop climate-resilient, salt-tolerant rice varieties.

The effectiveness of the protocol has been validated by successfully phenotyping a transgressive recombinant inbred population of IR29 x Pokkali for salinity tolerance at both IRRI fields in Los Banos and a farmer’s field in the coastal area of Infanta, Quezon, Philippines, using seawater irrigation (Kitazumi et al., 2024). This platform is not only a research tool but a strategic asset for salinity research and breeding programs aiming to deliver climate-resilient varieties to farmers.

## Practical considerations: infrastructure, cost, and operational efficiency

While acknowledging the infrastructure requirements of the platform, it is important to emphasize its highly economical and scalable design. The core infrastructure consists of simple, readily available materials: PVC pipes (96 units of 2 inch and 10 units of 6-inch), plastic water tanks (six large tanks (2000 liters capacity) and eight small tanks (1000 liters capacity), standard water pumps (12 hp), flexible hoses with gated inlets and outlets to control water flow (42 large PVC ball valves -110 mm and 92 small PVC ball valves -75 mm), 8 large PVC elbows (110 mm, 90°) and 92 small PVC elbows (75 mm, 90°) for directional changes, and 92 units of PVC T-connectors (75mm and 110mm). These materials are inexpensive, widely available in local markets, and require no specialized engineering for assembly. The total infrastructure cost for establishing a complete screening facility with 5–6 microplots per block is substantially lower than constructing and maintaining controlled-environment facilities such as greenhouses or growth chambers, which require climate control systems, hydroponic setups, and continuous energy inputs.

A critical advantage of this setup is the dramatic reduction in labor and time required for salt application. Under the traditional approach described in **Supplementary File S1**, one laborer can cover approximately 15–20 microplots per day, manually distributing salt granules and mixing them into the soil through puddling—a physically demanding and time-consuming process. In contrast, with the new saline-water irrigation system, the same 15–20 microplots can be irrigated with precisely calibrated saline water in approximately 1–2 minutes per microplot (**Figure 1**), representing a time reduction of over 95%. This efficiency extends to the entire experimental field: a complete field of microplots can be salinized within minutes rather than days, allowing a single operator to manage the entire facility with minimal physical effort. Moreover, the dissolved-salt approach eliminates the variability inherent in manual granule distribution, thereby improving both operational efficiency and phenotyping precision (https://www.irri.org/news-and-events/news/reinventing-salt-tolerant-rice-breeding-ground-zero).

We acknowledge that maintaining consistent salinity thresholds across diverse environmental conditions remains challenging. Factors such as ambient temperature, rainfall events, evapotranspiration rates, and soil composition can influence EC levels. However, the platform incorporates built-in mitigation strategies: piezometers installed in each microplot (4–6 per 36 m² plot) enable real-time EC monitoring; a flushing strategy using freshwater canals and inlets allows rapid EC adjustment when levels exceed target thresholds; and the row-column coordinate system facilitates *post-hoc* spatial correction through mixed-model analysis (Hussain et al., 2022). These features collectively ensure that salinity stress remains within the desired range (below 10 dS/m during the seedling stage, 12–15 dS/m during the reproductive stage) despite environmental fluctuations.

## Discussion and future outlook

This new field-based salinity screening platform marks a milestone in rice breeding—bridging the persistent gap between controlled research environments and the complex, variable realities of farmers’ fields. For the first time globally, a system has been developed that enables consistent and targeted salinity stress in the field conditions across the entire crop growth cycle, including both seedling and reproductive stages (**Figure 2**). This breakthrough allows for more accurate, reliable, and field-relevant evaluation of genotype performance, making it a game-changer in the pursuit of climate-resilient, salt-tolerant rice varieties.

A particularly groundbreaking component of this platform is the seawater irrigation facility established at IRRI—the first of its kind globally (https://bit.ly/3SQiJiP). This facility is not intended to grow rice in seawater, but rather to push the boundaries of phenotyping by exposing landraces and genebank materials to extreme salinity conditions. The goal is to uncover rare, high-value donors for breeding and genetic dissection—akin to finding a needle in a haystack. This approach enables the identification of genotypes with exceptional resilience, which would otherwise remain hidden under moderate or artificial screening conditions.

Looking ahead, the implications of this platform extend well beyond rice. Its modular, scalable, and cost-effective design makes it highly adaptable for other crops affected by salinity stress. By enabling high-throughput, field-relevant phenotyping, this setup lays the foundation for a new era of translational breeding—one that is grounded in environmental realism, driven by analytical precision, and focused on real-world impact. As climate change accelerates and salinity threatens global food security, this platform provides a robust and scalable solution to accelerate varietal development, enhance farmer resilience, and build more sustainable agricultural systems worldwide.
