# Supplementary material for: From labs to field realities: a paradigm shift in rice salinity screening
Source: Front Plant Sci. 2026 Apr 22;17:1812738. doi: 10.3389/fpls.2026.1812738 (PMC13147186; doi:10.3389/fpls.2026.1812738)
Supplement: Supplementary file 1 [file DataSheet1.docx]

**Supplementary File S1**

**Protocol 1: Artificial Salinity Field (ASF) Experiment at IRRI**

The Artificial Salinity Field (ASF) experiment at IRRI is a key platform for evaluating rice breeding lines under realistic, field‑based salinity stress conditions (**Fig. 1**). In contrast to controlled greenhouse or hydroponic screening systems, salinity stress in the ASF was imposed directly in the field using a tank‑and‑pipe irrigation infrastructure. Iodized white sea‑salt granules (Lot No. 233006) were dissolved in a centralized mixing tank to achieve the target electrical conductivity (EC) and subsequently applied uniformly to individual micro‑plots through a pipe‑based distribution network. The experiment was conducted in designated fields to minimize long‑term soil degradation and prevent residual salinity effects. By closely simulating salinity stress conditions typically encountered in farmers’ fields, the ASF enables the generation of robust and agronomically relevant phenotypic data, thereby supporting the reliable identification and selection of salt‑tolerant rice genotypes, despite the inherent challenges of maintaining uniform stress levels under variable field environments.


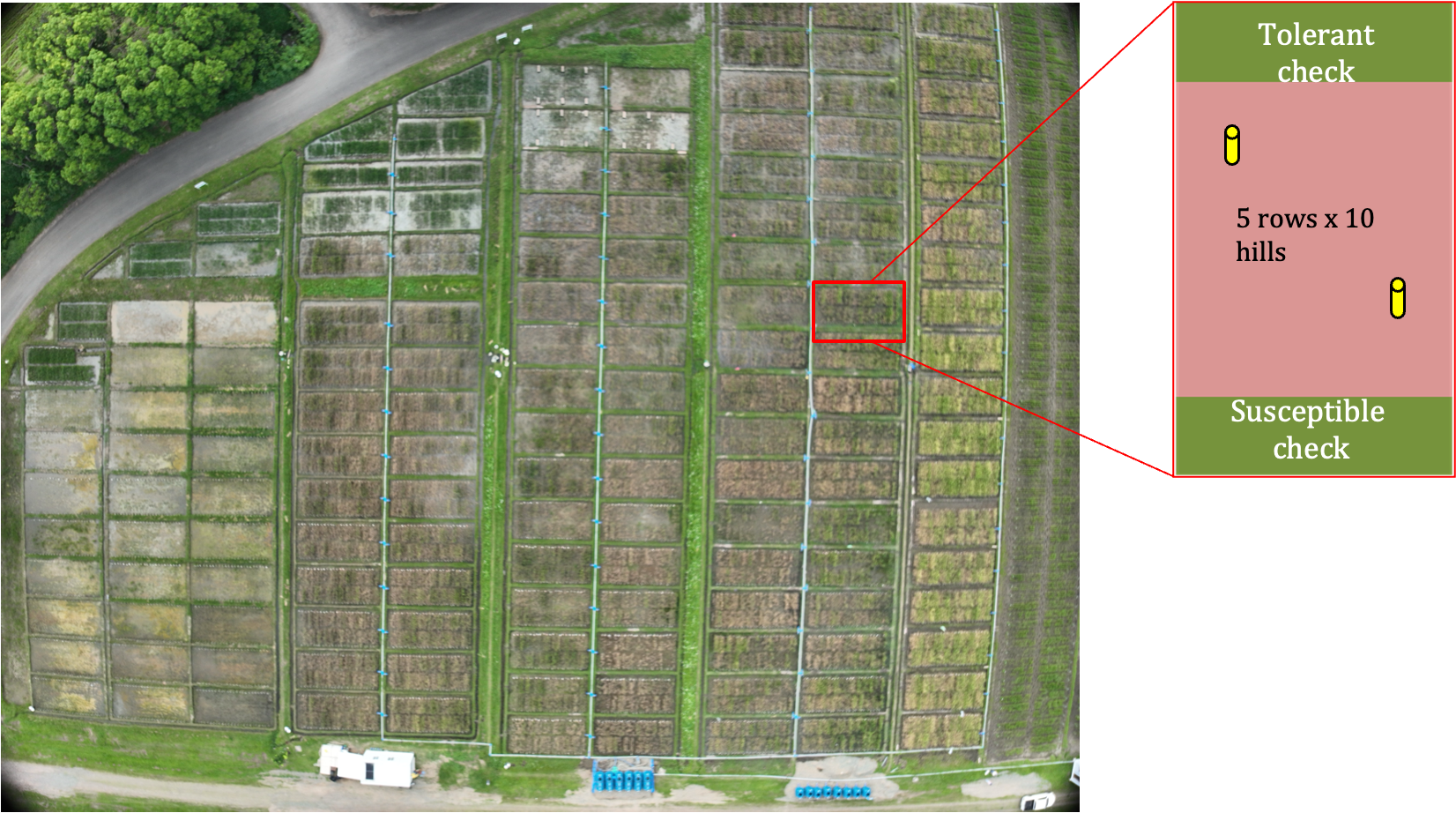


**Figure 1.** Artificial Salinity Field (ASF) experimental sites at IRRI HQ, Philippines. The yellow border outlines the entire experimental area, which is divided into multiple blocks. The red box highlights a representative micro-plot, each of which contains both test and check entries. Yellow tube structures indicate the locations of piezometers installed for monitoring salinity levels. Each micro-plot is monitored weekly (1–2 times) during the seedling and reproductive stages to assess electrical conductivity (EC) and ensure consistent salinity stress across the trial.

**Plant Materials**

The salinity field screening trial is conducted to assess a wide range of test entries alongside standard check varieties that serve as benchmarks for salinity tolerance and sensitivity. Salinity-tolerant checks include FL 478 (IR66946-3R-178-1-1), known for its seedling-stage tolerance; CSR 28 (IR51485-AC6534-4), a widely recognized tolerant line; IRRI 147 (IR63307-4B-4-3/NSIC Rc 182), a released variety with strong tolerance; IRRI 239 (IR91716-60-BAY-1-3-2/NSIC Rc 606); and IRRI 242 (GSR IR2-DQ25-L1-C1/NSIC Rc 612). Salinity-sensitive checks include IRRI 154 (IR04A421/NSIC Rc 222), representing high-yielding irrigated types; IRRI 119 (IR57515-PMI 8-1-1-SRN 1-1/PSBRc 68), which is tolerant to submergence and moderate flooding but sensitive to salinity; and the classic sensitive lines IR 29 (IR2061-464-4-14) and IR 42 (IR2071-586-5-6). All materials are freely available from IRRI under the Standard Material Transfer Agreement (SMTA). Additionally, local cultivars or breeding lines with known salinity tolerance should be included to enable comparative evaluation under specific environmental conditions.

**1. Trial planning**

Effective trial planning is critical to ensuring uniform, controlled salinity stress across the field. Proper land preparation should include the development of micro-plots to manage salinization and minimize spatial variability. Salinity levels are regulated using a combination of dissolved salts and standard irrigation practices, with drainage canals installed to remove excess seawater under severe stress conditions. The timing of stress application is crucial: from transplanting to active tillering, the field should maintain an electrical conductivity (EC) of 4–6 dS/m (**Fig. 2**). After tillering, saltwater irrigation should be used to raise the EC 10 to 15 dS/m until the heading stage. If EC levels become excessively high due to high stress intensity, fresh water may be applied to moderate salinity stress. This strategic approach ensures consistent stress exposure during critical growth phases, enabling accurate phenotypic screening for salinity tolerance.


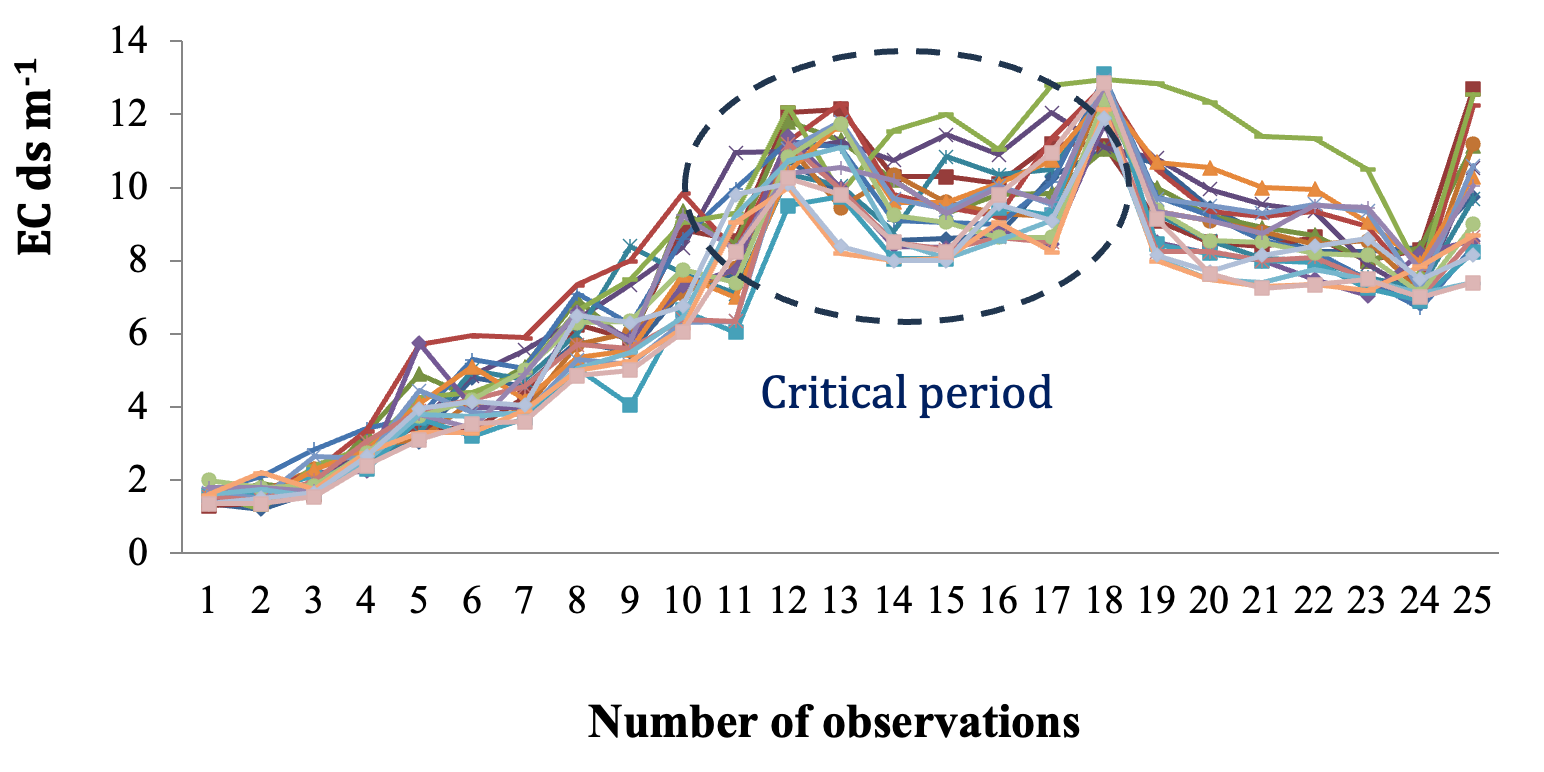


**Figure 2.** Salinity stress imposed from the early growth stage through to the reproductive stage across different plots, showing progressive increases in electrical conductivity (EC). The graphical representation illustrates the rise in EC levels during the reproductive stage.

1. **Field Design and Layout**

Field designs play a crucial role in evaluating breeding materials under salinity stress, ensuring results are both efficient and cost-effective while accurately identifying the most promising genotypes. Key design principles include replication, which helps create blocks to minimize plot-to-plot variation; randomization, which ensures unbiased estimation of means and variances; and careful management of plot variation to reduce error variance. Salinity screening plots are typically laid out in 10 m × 5 m rectangular formats, each bordered by drainage canals on two sides for effective water management. Depending on space availability, an Augmented Randomized Complete Block Design (RCBD) or an Alpha lattice design is recommended, with two replications if feasible. Within each plot, a spacing of 20 × 20 cm is maintained between rows and hills, with each plot ideally measuring around 5 m². To ensure statistical robustness and accurate stress validation, four local and eight international check varieties should be replicated within each block. Additionally, two rows of salinity-tolerant and -sensitive local checks should be strategically placed in the center and along plot borders or intermittently within the block. These serve as internal indicators to confirm the consistency of stress impositions. Fertilizer application should follow locally recommended rates to support uniform crop growth across treatments

- **Seedling Nursery Establishment**

To raise seedlings prior to transplanting, a well‑prepared nursery seedbed is essential. Each seedbed should be approximately 30 cm wide, with furrows spaced 10 cm apart to ensure uniform row alignment. Before sowing, clearly labeled tags should be placed across the seedbed, and seed envelopes—numbered according to the randomized trial layout—must be prepared at least one day in advance. For each three‑row plot (comprising approximately 25 hills per row within a 3 m² area), about 12 g of seed is required. Seeds should be placed in properly labeled envelopes and subjected to dormancy breaking by incubating at 50 °C for 3–5 days. Sowing can be carried out on either wet or dry seedbeds using line sowing in the prepared furrows, after which the seeds should be lightly covered with soil or fine garden soil to a depth of approximately 1 cm. During sowing, a basal application of 30–40g m⁻² of a complete NPK fertilizer is recommended. For effective weed management, a pre‑emergence herbicide should be applied 24–48 hours after the first irrigation. Throughout the nursery phase, consistent and adequate irrigation must be maintained to promote healthy seedling growth and ensure seedlings are suitable for timely transplanting.

1. **Land or Field Preparation**

Effective land preparation is essential to ensure optimal conditions for seedling establishment, particularly under salinity screening trials. Typically conducted about one month prior to transplanting, field preparation helps control weeds and improves soil fertility by incorporating crop residues from the previous season. Standard operations include plowing, puddling, harrowing, and leveling, tailored to the site-specific or farmer-recommended practices. The field must be leveled accurately—preferably using a wooden plank—to maintain minimal water levels, particularly in lowland rice systems. During preparation, fresh water should be used to flood and condition the soil, ensuring an electrical conductivity (EC) of 2–4 dS/m. Seawater intrusion, common in coastal areas during high tide, should be managed carefully by flushing and draining the field with fresh water to prevent high salt concentrations that may hinder seedling establishment. To support efficient water and salinity management and reduce field heterogeneity, two layers of compacted, mud-plastered bunds should be constructed around the perimeter, each measuring at least 30 cm in width and 15 cm in height. These allow water containment and ease of movement. Within the field, micro-plots should also be created based on the experimental layout, serving as blocks for trial design. The experimental layout should consider total entries, replications, and design type, with each plot measuring a minimum of 3 m² (accommodating 3 rows of 25 hills per row, spaced 20 × 20 cm). Bamboo sticks and printed tags should be used to clearly mark trial names, entry numbers, and plot identifiers. To properly monitor the effectiveness of salinity stress, two additional rows of known tolerant and susceptible check varieties should be established in the center and at the ends of plots. Finally, basal fertilizer must be applied during final land leveling according to farmer practice, and if necessary, molluscicide should be used to control snail infestation.

1. **Trial Establishment**

For effective field establishment, seedlings are pulled at around 21 days old and are carefully sorted and distributed according to their designated plot numbers. Prior to transplanting, excess water must be drained from the field to ensure adequate root-soil contact, which enhances establishment. Each hill should receive only one healthy seedling. Any extra seedlings should be retained for potential replanting, which must be done within seven days of transplanting to fill any gaps or missing hills efficiently. Proper weed management is essential throughout the trial. A pre-emergence herbicide should be applied one to two days after transplanting when the field has either no standing water or very minimal moisture. Post-emergence herbicide should be applied when weeds reach the 3–4 leaf stage. In addition, hand weeding or spot weeding should be performed as needed to maintain uniform competition-free conditions for plant growth. Fertilizer application should be carried out based on region-specific recommendations or standard farmer practices to support productive plant growth. To mitigate pest incidence, particularly from key rice pests, timely application of suitable insecticides is also necessary.

1. **Imposition of Salt Stress**

The induction of salt stress begins at approximately 42-45 days after sowing (DAS), when plants enter the active vegetative growth phase. The electrical conductivity (EC) of the soil water is gradually raised, reaching 10–12 dS/m during the tillering stage and increasing further to approximately 15 dS/m at the booting or panicle initiation stage (**Fig. 3**). This gradual and sustained salinization ensures that all entries, regardless of their duration or growth type, are uniformly exposed to salt stress across the trial period.

-
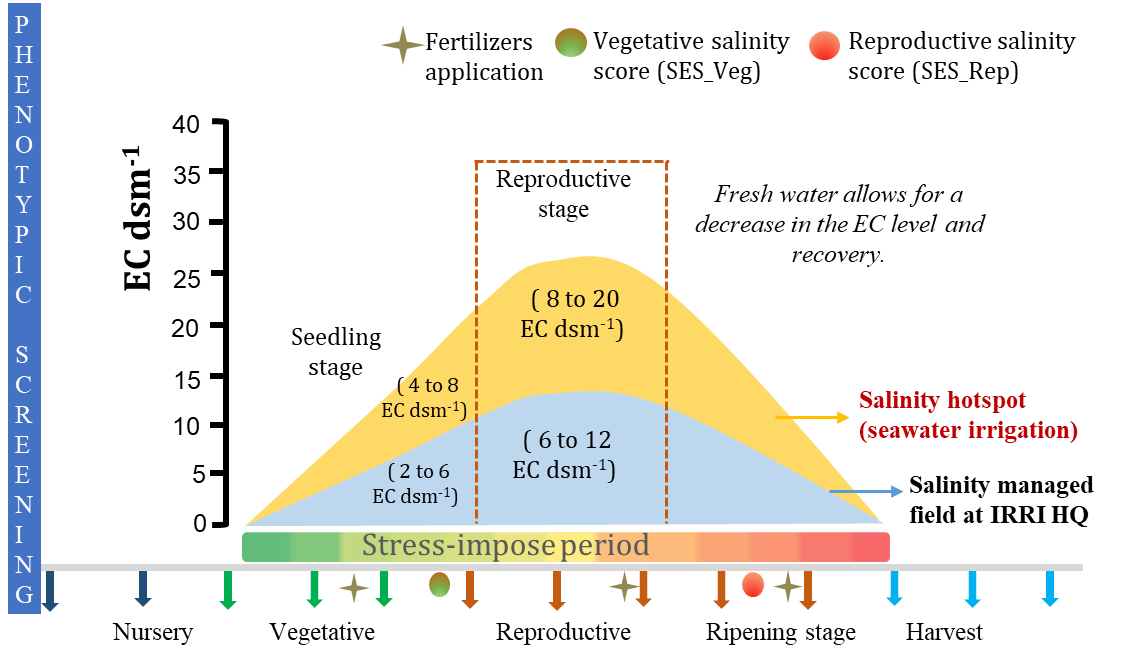
**Figure 3**: Crop growth period and imposing the saline stress in ASF at IRRI HQ. The targeted saline levels (EC 10 to 12 dS m−1) will be exposed at 60-day-old seedlings (at active tillering towards the booting stage/ panicle initiation stage). Once the targeted EC level reached the reproductive stage, it was maintained until 100 days from sowing. Further, the field is flushed with fresh irrigation water to wash off the salt in the soil. This process will also allow the recovery of the plants from salinity stress

To monitor salinity levels accurately, piezometers must be properly installed at two points per 10 m² plot. These piezometers should be cleaned and securely embedded in boreholes with collars sealed in mud to prevent leakage. An EC meter, calibrated regularly, is used to measure soil water conductivity, and measurements should be taken before and after salt applications. The water inside the piezometers must be pumped out prior to sampling, particularly before any new salt application. A pH meter is used in parallel to monitor the soil solution’s hydrogen ion concentration, and both EC and pH data should be recorded consistently throughout the trial period. A logbook must be maintained to track EC readings, pH values, and the precise volume or mass of salt applied per plot. Before initiating salinization, a baseline soil EC reading must first be established. Salinity is then imposed by dissolving 40 kg of commercial-grade NaCl (e.g., White Diamond Iodize Salt) in 2000 liters of fresh water using a mixing tanks targeting an initial EC of about 32-33 dS/m. The saline solution is applied uniformly across the micro-plots using the established piping distribution system, ensuring uniform distribution within each micro-plot. Application is carried out across the plot center, inter‑row spaces, and plot edges to promote homogeneous salt distribution within each plot. Special care is taken to ensure that both salt‑susceptible and salt‑tolerant check varieties receive comparable levels of salinity stress, thereby confirming the effectiveness and consistency of the salinity treatment across all experimental entries. The EC should initially be maintained at 2–4 dS/m during transplanting and gradually increased to 8-10 dS/m between early vegetative and tillering stages. As the crop progresses toward maximum tillering and the reproductive stages, EC should be raised to 15 dS/m and maintained until about 100 DAS. If signs of excessive or early stress—such as severe leaf burning or browning—are noticed, a fresh water flush may be necessary to reduce EC levels and permit plant recovery. This "desalination" flush also helps normalize field conditions after the stress period, enabling researchers to evaluate grain yield and other post-stress agronomic responses. Regular irrigation with fresh water following the stress period further aids in leaching excess salts from the root zone.

All genotypes are carefully monitored throughout the cycle for visible signs of salt injury. These symptoms are scored using the IRRI Standard Evaluation System (SES, IRRI 2015), allowing for consistent rating of tolerance levels across genotypes.

1. **Data Collection**

Accurate data collection is crucial for evaluating genotype responses to salinity stress. Basic site data, including soil type, elevation, slope, geographic coordinates, temperature, and rainfall records, should be documented. Management practices that differ from the standard procedure should also be recorded to explain trial variations. Traits to be measured include visual salt injury scores at vegetative and reproductive stages using the IRRI –modified Standard Evaluation System (SES), flowering date when 50% of the plants in a plot have flowered, plant height from three randomly selected plants per plot, grain yield in grams, the number of hills harvested, and the grain’s moisture content. Missing values should also be documented per trait and per replication to ensure complete and rigorous analysis.

This protocol ensures consistency, reliability, and comparability in evaluating rice genotypes or other crops for salinity tolerance under managed field conditions. It is based on best practices established by the International Rice Research Institute (IRRI) and can be adapted regionally based on resource availability and environmental considerations.

**Protocol 2: Natural Salinity Field (NSF) Experiment at The Coastal Hotspot Location**

The NSF experiment is conducted at a coastal hotspot site characterized by naturally occurring salinity conditions, providing a realistic environment for evaluating rice genotypes at the field level using seawater irrigation (**Fig. 4**). This location enables the assessment of genotypic performance across growth stages under fluctuating salinity levels influenced by tidal patterns and seasonal variations. The field is divided into micro-plots to manage spatial variability and ensure consistent exposure to salinity stress. Electrical conductivity (EC) is regularly monitored, with natural salinity levels typically ranging from 4–6 dS/m during early growth stages and exceeding 15 dS/m during the reproductive phase. At certain times, it may increase more than 20 due to high tides in coastal regions. The use of local water sources, including brackish and tidal water, simulates real-world stress conditions. This setup enables the identification of promising genotypes with stable performance under natural salinity, supporting breeding efforts for salt-tolerant rice varieties adapted to coastal agroecosystems.


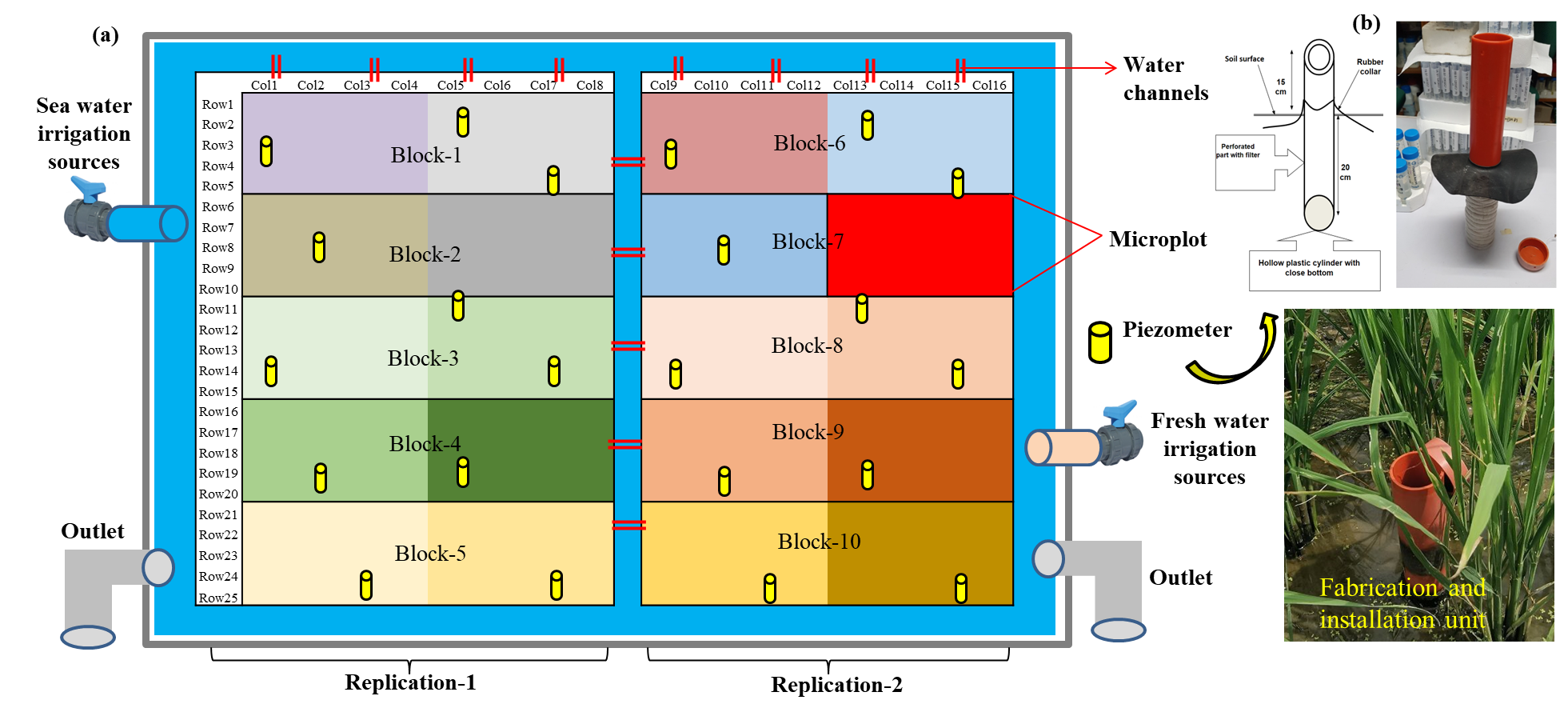


**Figure 4.**  Field layout design for salinity phenotypic screening facility in coastal regions. (a) An example of the layout shows the number of tested entries divided into 20 micro-plots in both replications (a pictorial diagram representing the 200 test entries, using the alpha lattice design method with two replications) across a total of 20 blocks. Each block consists of 20 entries, and the piezometers were installed across all blocks to measure stress intensity levels in the field. Monitoring the EC levels in both the replications was installing the Piezometer randomly. Blue represents the seawater irrigation across the field through well–connected channels, and excess water can be removed from the outlets. (b) A detailed Piezometer structure to capture the field's salinity stress levels.

1. **Seedling Nursery**
   1. Seeds per entry are prepared into properly labeled envelopes. 12 g of seed is enough for 3-row x 25 hills with 20cm x 20cm spacing or 3 square meters plot size.
   2. Break seed dormancy by placing the seeds into the oven set at 50⁰C for 3-5 days.
   3. Prepare wet beds or dry beds with furrows one day before sowing.
   4. Apply complete NPK fertilizer (14-14-14) at 30-40 grams per meter row

- Sow the seeds in properly labeled furrows. The one-row gap can be kept between rows to avoid mixing and promoting healthier growth. Cover seeds with fine garden soil by at least 1 cm, ensuring all the seeds are properly covered.
  1. Spray pre-emergence herbicide 24-48 hours after sowing.
- Let the seedlings grow for 25-30 days with proper irrigation.
  1. It is recommended that the nursery bed is situated in a non-stress field to allow the normal growth of the seedlings.

1. **Field preparation**
   1. Initiate land preparation according to standard land preparation in the country. It is important to ensure the field is properly leveled before transplanting.
   2. Construct levees with at least 30 cm width and 15 cm depth (height) to contain the irrigation of every plot. Whenever possible, design the levees into two layers. The outermost layer of the bund has a lining of high-grade plastic sheets to contain the horizontal movement of the saline water to nearby normal plots. And the second layer can be the usual soil levees.
   3. Layout the field a day before pulling seedlings. Bamboo sticks and labeled tags are needed to identify plots of correct entries. Distribute tags on seedling rows. The plot size is 3 rows x 23-25 hills with 20cm x 20cm between and within row hill spacing.
   4. Before transplanting, collect the soil samples (at least three replicates per site), air-dry them for 72 h, expose them to sunlight, and crush them for laboratory analysis.
2. **Trial establishment and management**
   1. Carefully pull 25-30 day-old seedlings and transplant 1 seedling per hill into properly labelled plots.
   2. Apply locally recommended fertilizer rates.
   3. Apply pre-emergence herbicide 24-48 hours before transplanting. Post-emergence herbicide can be applied 10 days after transplanting. After applying the post-emergence herbicide, spot weeding is necessary to remove drop seeds and stubborn weeds.
   4. Maintain proper irrigation with standing water. Make sure to install Piezometer (see Fabrication and Installation of Piezometer for salinity trials) 2 sets per 10 sqm area, this will allow us to access the EC level across the fields.
   5. Before the EC reading, pump out the water first this will remove any clogging in the piezometer.
   6. Follow integrated insect pest and disease management practices.
   7. Allow the seedlings to grow and suffer from saltwater intrusion. In case the salinity stress is very high and fresh water is available, flush or irrigate the field with fresh water to flush out excess salt. If fresh water is unavailable, drain the field to remove excess water in the field.
   8. For rice fields near coastal areas, allow seawater to enter the experimental field. Several entry points in strategic areas in the levees/ bunds should be open to allow seawater intrusion into the field. This will help for an even distribution of seawater to the field. Sea water intrusion is easier when there is high tide, but during low tide, it is necessary to use water pumps to help seawater to enter the field. If, in any case, the EC level is too high, irrigate the field using fresh water. But if no fresh water is available, drain the excess seawater out of the field. Maintain EC level to 15 dS/m until 100 DAS, then flushing or irrigating with fresh water to desalinize the field. If, in any case, there is a need for several saltwater intrusions, you may do so just while making sure to properly record all relevant information in the field book (date when the seawater intrusion happen and EC reading of the experimental field after the seawater intrusion).
   9. Maintain the EC 4-6dS/m from transplanting to the maximum tillering stage. Maintain the EC of 10-15 at initial panicle initiation to the heading stage. Since seawater intrusion may come anytime during high tides, ensure to get and record the EC after every seawater intrusion to monitor the level of EC of the field trials. Irrigation with normal/fresh water can be done if the EC of the field is very, very high, and the crop is damaged.
   10. At 100 DAS, we can assess the field if we need to stop the seawater intrusion or not. If the damage is severe, we start desalinization of the field by irrigating with fresh water and allow the plants to recover the yield data per plot.

**Note:** *In scoring a salinized trial, it is essential to carefully look at the plants as there are entries that may score low but could give good grain filling and low spikelet sterility despite severe leaf burning. For salinity field ( natural salinity field), transplant 30 day old seedlings to ensure good seedling recovery after transplanting.*

1. **Fabrication and Installation of Piezometer for Salinity Trials: Materials:**
2. 30cm-long × 2-inch-diameter PVC pipe
3. drill or hacksaw
4. rubber collar
5. porous cloth
6. PCV cap

The EC meter measures the amount of electrolytes present in the solution. The amount of electrolytes is associated with the degree of salinity. However, calibration of the EC meter should be done at least once a month using the 7.01 dS m" buffer. NaCl granules used for the ASF is a White IodizeSea Salt, Artemis Salt Corporation, 50 kg - food grade, iodized granulated salt. At least 2 pieces of piezometers are installed every 10sqm plot to ensure we can capture the readings uniformly across the different micro-plots. The EC meter measures the amount of electrolytes present in the solution. The amount of electrolytes is associated with the degree of salinity. However, calibration of the EC meter should be done at least once a month using the 7.01 dS m" buffer. The EC and pH of the field can be measured from the water seeped into the piezometer. EC readings should be monitored regularly (piezometer and standing water).

1. **Data Collection**

In the coastal system under salinity stress, the major breeding activities include phenotypic performance of stage-specific growth scores at vegetative and reproductive stages (SES score; **Table 1**), grain yield, maturity, and other preferred agronomic traits, which must be collected. The genotype performance's overall phenotypic acceptability needs to be recorded two weeks before harvest. Harvest the entire plot, except for the front and back rows, to avoid border effects on yield. Remember to record the number of hills harvested for each plot and the harvest date.

- Harvested samples should be appropriately labelled using the printed harvested tags, which should at least include the trial number and plot number. The harvest tag should match the field tag corresponding to the plot, and both tags should be placed inside the net bag during harvest.
- Harvested samples should be placed inside the oven for drying at 50 °C for 2-3 days and then threshed using the appropriate thresher machine.
- Threshed samples should be cleaned with a seed blower, and the cleaned seeds must be placed in the paper bag with proper labels.
- Properly labeled paper bag. The printed harvest tag should also be placed inside the paper bag.

**Data record**

Accurate and systematic data recording is critical to the success and reproducibility of any field experiment, particularly in salinity screening trials. Below is an effective approach for documenting all relevant agronomic, environmental, and management data, organized for clarity and ease of analysis.

- **Sowing Date:** Document the exact day seeds are first sown in the nursery or field.
- **Transplanting Date:** Record when seedlings are transplanted from the nursery to the main field plots.
- **Number of Rows Per Plot:** Note the total number of rows established within each experimental plot.
- **Number of Plants Planted Per Row:** Specify the number of individual plants set out in each row.
- **Intra-Row Spacing (cm):** Measure and record the distance between plants within the same row, in centimeters.
- **Inter-Row Spacing (cm):** Measure the space between adjacent rows, ensuring uniformity across plots.
- **Plot Size (m²):** Calculate and record the area of each plot in square meters.
- **Fertilizer Application:** Detail all types and dosages of fertilizer applied, including the exact dates of each application.
- **Days to 50% Flowering:** Record the number of days from transplanting until 50% of the plants within a plot to have reached flowering.

**Plant Height (cm):** Record the height from root tip to the longest leaf tip for selected plants at the appropriate growth stage, both in field and tube experiments$Plant height (HT\_AVG) =(HT1+HT2+HT3)/3$

Where: HT1, HT2, and HT3 are the heights of the three individual plants measured (usually in centimetres). This average gives a representative value for plant height per plot or genotype under evaluation

- **Number of Tillers per Plant:** Count all productive and non-productive tillers per plant at harvest in field conditions.
- **Grain yield:** To ensure accurate grain yield comparisons, exclude border rows from harvest. Include all hills affected by common pests and diseases, except those completely destroyed by rats or birds. Record the number of hills harvested per plot. After harvesting, thresh, dry, clean, and weigh the grains using a precision balance, expressing the weight in grams. For consistency, use oven-dried grains to standardize moisture content. Finally, calculate grain yield per plot as the net plot yield, adjusting for effective area and moisture content as needed

$$Grain yield (Kg/ha)=(GYT*((100-moisutre content)/86)/Effective area*10)$$

*Grain yield = weight of harvested grain (in grams)*

*86 = standard factor to adjust to 14% moisture content*

*10,000 = to convert square meters to hectares*

*Effective Area = net harvested plot area (in m²)*

*Moisture Content = measured grain moisture at harvest (in %)*

**Table 1 Salinity Severity Score (IRRI 2015):** Assign standard evaluation scores for salinity damage at both vegetative and reproductive stages using established guidelines.

| Score | Symptom/observations at vegetative stage | Degree of tolerance |
| --- | --- | --- |
| 1 | Normal growth, only the old leaves show white tips while no symptoms on young leaves | Highly tolerant |
| 3 | Near normal growth, but only leaf tips burn, few older leaves become whitish partially and rolled | Tolerant |
| 5 | Growth severely retarded; most leaves severely injured, few young leaves elongating | Moderately tolerant |
| 7 | Complete cessation of growth; most leaves dried; only a few young leaves still green | Sensitive |
| 9 | Almost all plants dead or dying | Highly sensitive |
| Score | **Symptom/observation for Reproductive stage** | **Degree of tolerance** |
| 1 | Healthy growth, spikelet sterility at ≤5% | Highly tolerant |
| 3 | Growth slightly stunted, spikelet sterility at > 5%-20% | Tolerant |
| 5 | Growth moderately stunted, ¼ of all leaves brown, panicles partially exerted, spikelet sterility at 21%-40% | Moderately tolerant |
| 7 | Growth severely stunted with about ½ of all leaves becoming brown, panicles poorly exerted, high sterility at 41%-70% | Sensitive |
| 9 | Growth severely stunted with almost all the leaves becoming brown and affected, panicles not exerted, delayed heading, or papery florets / chaffy panicles with very high sterility at >70% | Highly sensitive |
